# Colistin exposure enhances expression of *eptB* in colistin-resistant *Escherichia coli* co-harboring *mcr*-1

**DOI:** 10.1038/s41598-022-05435-0

**Published:** 2022-01-25

**Authors:** Rajkumari Elizabeth, Somorita Baishya, Bubul Kalita, Jayalaxmi Wangkheimayum, Manabendra Dutta Choudhury, Debadatta Dhar Chanda, Amitabha Bhattacharjee

**Affiliations:** 1 Department of Microbiology, Assam University, Cachar, Silchar, 788011 India; 2 Department of Life Science and Bioinformatics, Assam University, Silchar, India; 3 Department of Microbiology, Silchar Medical College & Hospital, Silchar, India

**Keywords:** Antimicrobial resistance, Bacteriology

## Abstract

Colistin resistance has increased due to the increasing and inappropriate use of this antibiotic. The mechanism involves modification of lipid A with phosphoethanolamine (PEtN) and/or 4-amino-4deoxy-l-arabinose (L-Ara4N). *EptA* and *eptB* catalyze the transfer of phosphoethanolamine to lipid A. In this study, gene network was constructed to find the associated genes related to colistin resistance, and further in vitro validation by transcriptional analysis was performed. In silico studies showed that *eptB* gene is a highly interconnected node in colistin resistance gene network. To ascertain these findings twelve colistin-resistant clinical isolates of *Escherichia coli* were selected in which five were harboring the plasmid-mediated *mcr*-1. Screening for colistin resistance was performed by broth microdilution (BMD) method and Rapid polymyxin NP test. PCR confirmed the presence of the *eptA* and *eptB* genes in all isolates and five isolates were harboring *mcr*-1. Transcriptional expression in five isolates harboring *mcr*-1, showed an enhanced expression of *eptB* when exposed under sub-inhibitory colistin stress. The present study for the first time highlighted genetic interplay between *mcr*-1 and *eptA* and *eptB* under colistin exposure.

## Introduction

Polymyxins such as polymyxin B and colistin (polymyxin E) are utilized as the last therapeutic option due to the emergence of multidrug-resistant bacteria mainly against carbapenem-resistant Gram-negative bacteria including Enterobacteriaceae^1^. Resistance to colistin has increased steadily due to the increasing and inappropriate use of this antibiotic^2^. The mechanism of colistin resistance in Gram-negative bacteria is associated with specific modification of lipid A with phosphoethanolamine (PEtN) and/or 4-amino-4deoxy-l-arabinose (L-Ara4N)^3^. The synthesis and addition of L-Ara4N to lipid A is stimulated by the arnBCADTEF operon. This operon is activated due to the mutation in the two-component systems (TCSs), mainly PhoPQ and PmrAB^4^. Representatives of PEtN transferases namely *eptA* and *eptB* are involved in the synthesis and addition of PEtN^5^. They are regulated by the PmrAB TCS, which itself can be upregulated by PhoPQ^4^. *EptA* catalyzes the transfer of PEtN from phosphatidylethanolamine (PE) onto the lipid A of Lipopolysaccharide (LPS) and *eptB* codes for Kdo (2)-lipid A phosphoethanolamine 7″-transferase which catalyzes the addition of a pEtN moiety to the outer 3-deoxy-d-manno-octulosonic acid (Kdo) residue of a Kdo (2)-lipid A. Phosphatidylethanolamines with one unsaturated acyl group functions as pEtN donors and the reaction releases diacylglycerol^6^. These LPS modifications reduced the negative charge of LPS thereby decreasing the affinity of LPS to positively charged colistin leading to resistance^7^. The plasmid-mediated PEtN transferase encoded colistin resistance gene, *mcr*-1, which is horizontally transferable was first described in China in 2015^8^. Since then, different types of *mcr* genes namely *mcr*-1 to *mcr*-10 have been identified and reported from different parts of the world^9^.

Thus, it is of immense importance to understand the associated gene networks of these resistance determinants and how they respond when bacteria are exposed to colistin pressure. This study involves an in silico and in vitro investigation of *eptA* and *eptB* and their expressional pattern within *E. coli* co-harboring *mcr*-1.

## Results

### Protein–protein interaction (PPI) analysis

**Figure 1:**
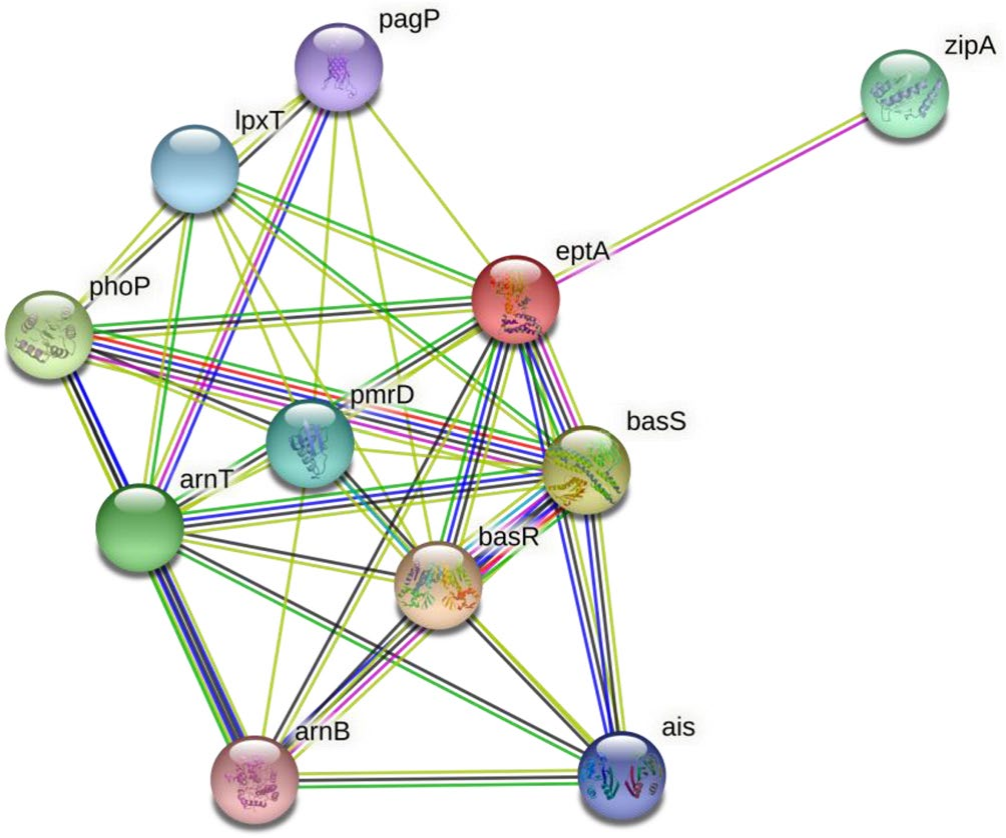
Functional partners associated with *ept*A.

**Figure 2:**
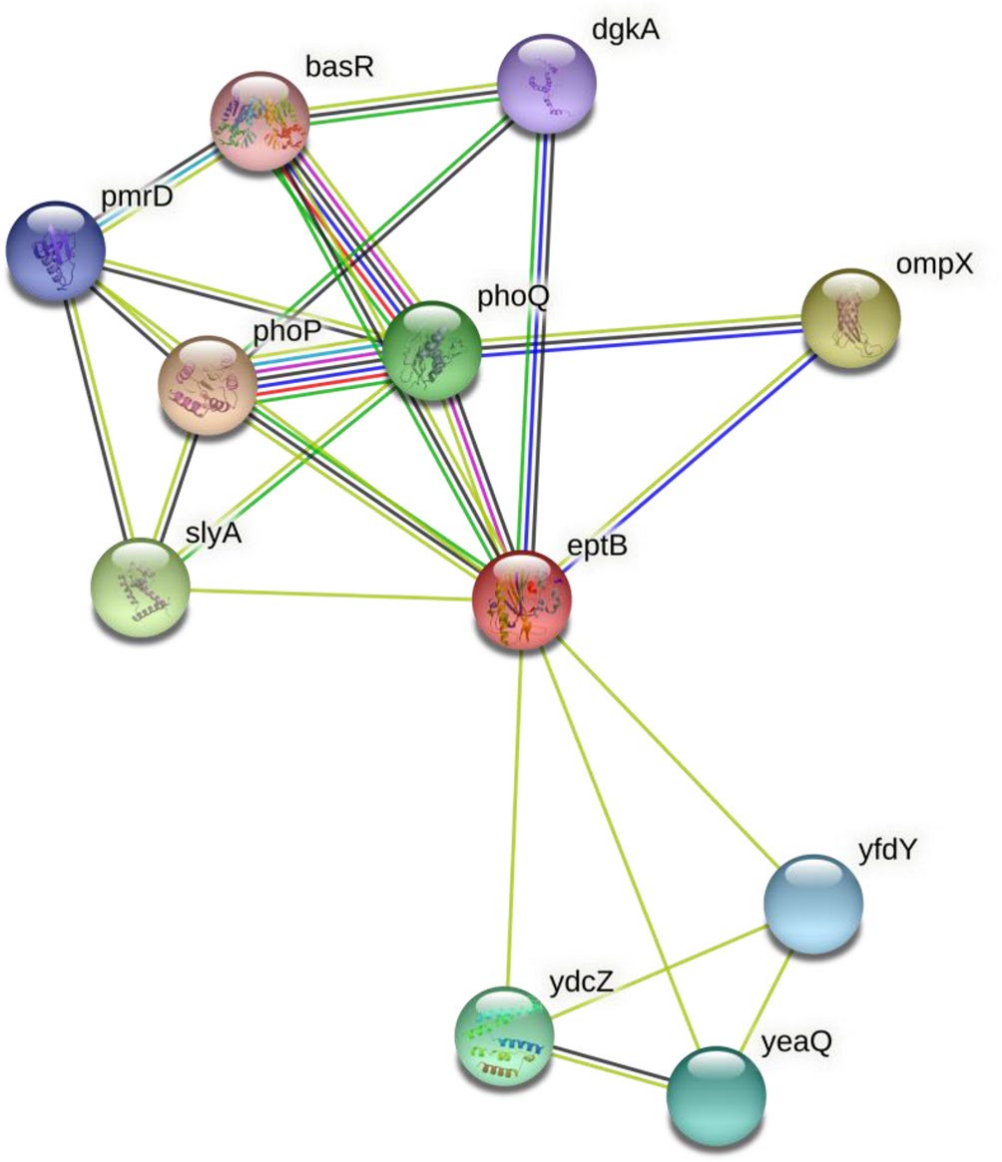
Functional partners associated with *ept*B.

STRING database generated PPIN of *eptA* and *eptB* genes. The network generated from this interaction showed that gene *eptA* interacted with 39 functional partners in total, i.e. at medium, high, and highest confidence levels *eptA* gene interacted with 31, 6, and 2 functional partners respectively. Similarly, PPIN of *eptB* gene showed that *eptB* interacted with 41 functional partners i.e. at medium and high confidence level 37 and 4 functional partners were associated respectively. No interactions were found at highest level for *eptB* gene. These confidence levels indicated the likeliness of the interaction based on evidences mentioned above. Functional partners associated with *eptA* and *eptB* genes are depicted in Figs. 1 and 2.

### Construction of gene network

**Figure 3:**
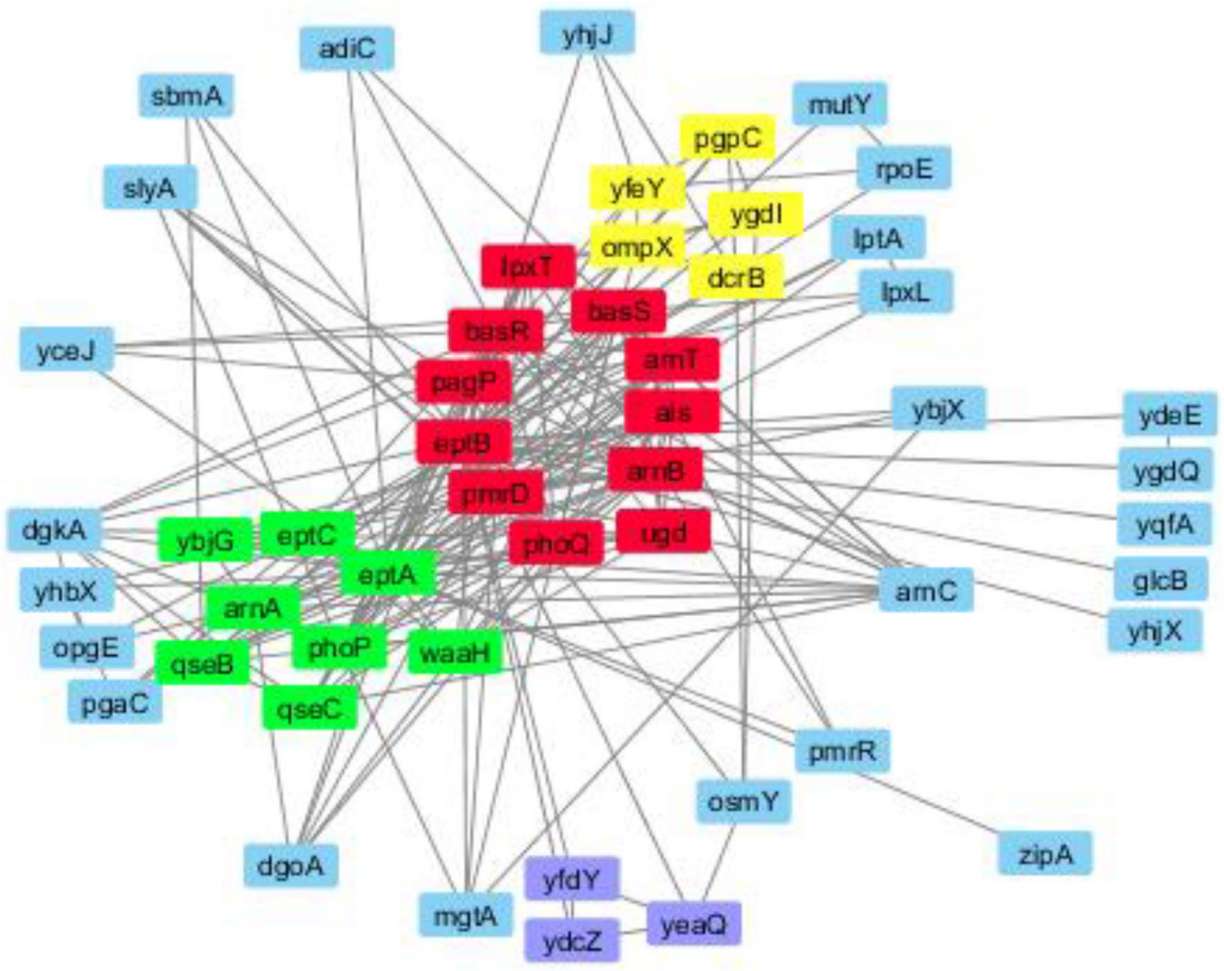
Gene network through which *ept*A and *ept*B genes interact.

Incorporation of these PPIN onto Cytoscape v 3.4.0 generated a gene network of 52 nodes and 210 edges (interaction). Figure 3 shows the network of genes through which *eptA* and *eptB* genes interact to impart colistin resistance.

### Analysis of cluster

Upon integration of PPIN into Cytoscape v 3.4.0, MCODE was employed for identifying the highly interconnected nodes. MCODE revealed 4 clusters with 27 nodes which were ranked based on the app’s algorithm. The rest of the nodes remained unclustered, hinting that they don’t have an active/influential role in imparting resistance. The cluster with the highest MCODE score had 11 nodes and 44 edges (Supplementary Fig. S1).

### Analysis of topological parameter

Topological properties such as average shortest path length and betweenness centrality of all the functional partners participating in the network were measured for the identification of the most influential genes of the network. It was observed that the *eptB* gene from cluster 1 had a significant influence on the network (Supplementary Fig. S2).

### Functional and pathway enrichment analysis

Functional and pathway enrichment analysis performed using STRING and ClueGO gave insights into the pathways associated with *eptA* and *eptB.* The significant terms associated with BP and MF (Molecular Functions) are depicted in Supplementary Fig. S3. In BP ontology, 4 enriched specific clusters with 36 GO terms were generated which were—lipopolysaccharide metabolic process (25 terms), intracellular signal transduction (13 terms), oligosaccharide biosynthetic process (14 terms), and response to iron ion (5 terms).

After analyzing the various properties of the selected genes involved with colistin resistance, it was found that the *eptB* gene from cluster 1 has a strong influence on the network owing to its high frequency in the network. Furthermore, *eptB* gene was found to be associated with 26 biological processes in the network which were part of the 3 major categories of the network-lipopolysaccharide metabolic process, intracellular signal transduction, oligosaccharide biosynthetic process. After all these analysis it is observed that *eptB* gene is a highly interconnected node (gene) of this network and can be predicted that this gene might play a crucial role in colistin resistance The processes and function with which the *eptB* gene is associated are given in Supplementary Table ST1.

### Screening of colistin-resistant *Escherichia coli*

Broth microdilution method and polymyxin NP test confirmed twelve *E. coli* isolates to be screened positive for colistin resistance. Genotypic characterization by PCR analysis revealed the presence of *eptA* and *eptB* genes in all the *E. coli* isolates. The *mcr*-1 gene was found to be horizontally transferable and carried by FIA Inc group plasmid.

### Plasmid elimination assay

Plasmids harboring colistin resistance determinants were successfully eliminated after a single treatment with sodium dodecyl sulfate (SDS; 4% to 10%).

### Transcriptional analysis

**Figure 4:**
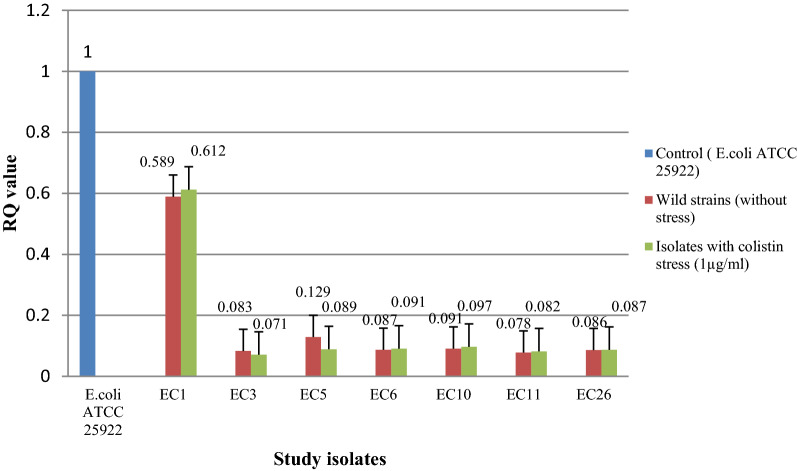
Transcriptional analysis of *ept*A for *mcr*-1 negative isolates.

**Figure 5:**
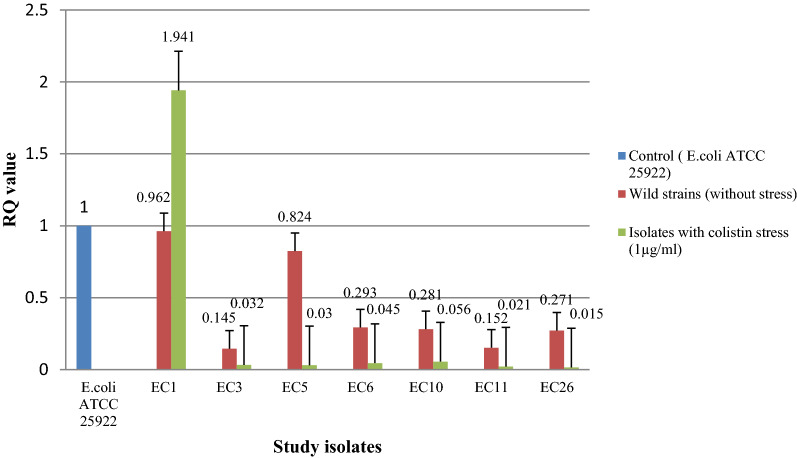
Transcriptional analysis of *ept*B for *mcr*-1 negative isolates.

**Figure 6:**
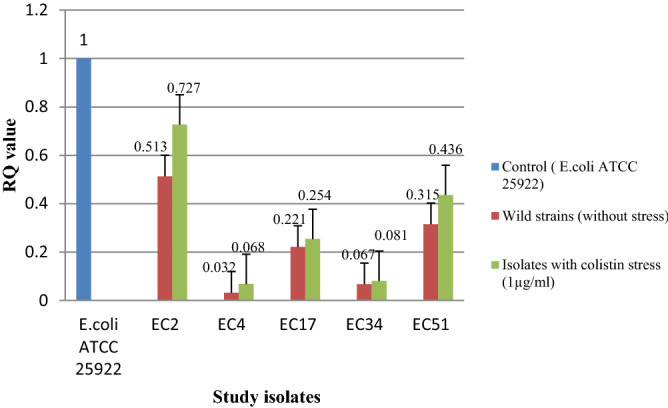
Transcriptional analysis of *ept*A for *mcr*-1 positive isolates.

**Figure 7:**
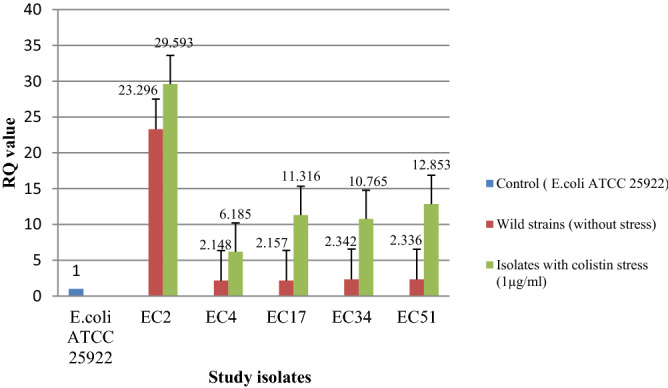
Transcriptional analysis of *ept*B for *mcr*-1 positive isolates.

Transcriptional expression pattern in seven isolates (devoid of any *mcr* gene) did not show any change in expression of *eptA* and *eptB* with and without colistin stress (Figs. 4 and 5). However, the expression level [RQ (Relative quantification) value] was higher for both *eptA* and *eptB* in five isolates (EC2, EC4, EC17, EC34, and EC51) harboring *mcr*-1 i.e., an acquired pEtN gene. An enhanced expression was observed for these five isolates for both the genes when exposed under the sub-inhibitory concentration of colistin (Figs. 6 and 7) (Supplementary Table ST2). Similar pattern of expression was observed for all the transformants harboring *mcr*-1 gene (Supplementary Figs. S6 and S7). However, for cured mutants, colistin stress did not enhance expression of *eptB* significantly (Supplementary Figs. S4 and S5).

## Discussion

The extensive use of colistin in hospitals and also as a growth promoter in poultry, agriculture, etc. has led to increased resistance to this drug. Case reports and outbreaks have been reported from around the world^10,11^. In India, colistin resistance has been reported in various clinical isolates, environmental isolates, and also isolates from food samples^11–13^. The chromosomally encoded intrinsic resistance to colistin occurs naturally due to the expression of certain genes such as *eptA* and *eptB* and the transferable colistin resistance is mediated by plasmid-borne *mcr* genes^14^. *EptB* gene encodes Ca^2+^ induced pEtN which modifies the outer Kdo residue of *E. coli* LPS, thus leading to colistin resistance. However, extensive studies regarding this gene are lacking^6^. Therefore, this study was undertaken to understand the role of *eptA* and *eptB* in imparting colistin resistance.

In this study, the role played by *eptA* and *eptB* in imparting colistin resistance was studied following an in silico approach. Gene network constructed using Cytoscape software showed that the *eptB* gene was a highly interconnected node in colistin resistance gene network. Analyzing various parameters of the gene network it was found that this gene had higher frequency in the network and was also associated with 3 major biological processes, which included lipopolysaccharide metabolic process, intracellular signal transduction, and oligosaccharide biosynthetic process.

To further ascertain their role, transcriptional analysis of *eptA* and *eptB* gene in colistin-resistant *E. coli* was performed. An induced expression of *eptB* in the *mcr*-1 positive resistant isolates on exposure to colistin was observed. This could be related to the previous studies that showed the induced activation of chromosomally mediated colistin resistance genes like *phoP*, *phoQ* in the presence of colistin^15,16^. Another study from Spain mentioned the close link between the *dgkA* gene with *mcr*-1 and *mcr*-3. The same study found that the expression of *mcr* determinants downregulated endogenous genes involved in LPS modification or phospholipid recycling, although to different extents of repression: strong for *arnB*, *ybjG*, and *pmrR*; medium for *eptA*^17^. In our study, overexpression of *eptB* was observed in the isolates which harbor the *mcr*-1 gene while a lower expression level was observed in the isolates which were devoid of *mcr*-1. This finding showed that the presence of *mcr*-1 might augment the expression of the *eptB* gene in the presence of colistin thereby conferring resistance. Additionally, colistin stress can enhance expression of endogenous genes (*eptA* and *eptB*) when *mcr*-1 is present within an isolate. Although *mcr*-1 product alone can confer colistin resistance, other regulatory factors must be involved in triggering expression of *eptA* and *eptB*.

Therefore, we might consider that *eptB* plays a crucial role in mediating colistin resistance by the *E. coli* isolates. This study data revealed that *eptB* mediated mechanism of colistin resistance persists in the clinical *E. coli* isolates of this area and the association of *mcr*-1 in enhancing the expression of *eptB* in the presence of sub-inhibitory concentration of colistin pose a striking impact in the scenario of resistance mechanisms to colistin in clinical *E. coli*. Thus, the present study for the first time highlighted genetic interplay between *mcr*-1 and *eptA* and *eptB* under colistin exposure.

## Materials and methods

### Construction of protein–protein interaction network (PPIN)

To decipher the relationship between genes and proteins associated with *eptA* and *eptB* genes of *E. coli* protein–protein interaction network (PPIN) was studied using STRING (Search Tool for the Retrieval of Interacting Genes/ Proteins, v 11) database and the results were incorporated into Cytoscape v3.4.0 software. The database generated a network by integrating known and predicted associations based on multiple evidences which were individually scored and color-coded. This evidence included interactions obtained by curating databases, text mining from scientific publications, and high and low throughput experiment assays. Protein identifier or protein sequence was required as query input and functional partners associated with the genes under consideration were generated based on the evidence at four confidence scores—highest (0.900), high (0.700), medium (0.400), and low (0.150)^18^.

### Construction of gene network

Cytoscape v 3.4.0, an open-source software package, was used for the visualization of the gene network. The software considers various parameters and helps in the visualization, modeling, and analysis of gene networks^19^.

### Analysis of strongly associated cluster

To identify the highly interconnected regions i.e. clusters of the network, the MCODE (Molecular Complex Detection) app from Cytoscape software was employed. Clusters were scored and ranked based on an algorithm that stressed identified the locally dense regions of the network. Parameters like degree cutoff = 2, node score cutoff = 0.2, k-core = 2 and max. depth = 100 were set as cut-off^20^.

### Analysis of topological parameters

Network Analyzer plugin of Cytoscape software was utilized for analysis of topological parameters that gave insights into small-world effect by considering properties such as average shortest path length and betweenness centrality. Betweenness centrality calculates the frequency of occurrence of a node (gene) through the shortest paths in a network, thus indicating the centrality of the node. The average shortest path length, on the other hand, estimates the shortest distance between the nodes^21^.

### Functional and pathway enrichment analysis

Functional enrichment analysis done using STRING v 11 and ClueGO app of Cytoscape gave gene ontological (GO) annotations of the genes involved in the network. GO terms for BP (Biological Processes) were generated based on a p-value cut-off ≤ of 0.05 for the functional partners^18,22^.

### Clinical isolates

A total of 291 consecutive, non-duplicate *E. coli* isolates were obtained from the Department of Microbiology, Silchar Medical College and Hospital (SMCH), Silchar. Twelve colistin-resistant *E. coli* isolates were selected for the study in which five among them were harboring *mcr*-1. The organisms were identified using VITEK^®^ 2 compact system (Biomeriux, USA). *E. coli* ATCC 25922 was used as a control.

### Screening of the isolates

The isolates were screened for colistin resistance by performing broth microdilution (BMD) method using colistin (Hi-Media, Mumbai, India) with a range from 2 to 4 μg/ml and the results were interpreted as per EUCAST guidelines 2017^23^. Additionally, Rapid polymyxin NP tests were also performed by using a defined concentration (5ug/ml) of colistin to further screen colistin resistance among the test isolates^24^.

### Horizontal transferability of colistin resistance

Purification of the plasmids of *mcr*-1 positive isolates was carried out by QIAprep Spin Miniprep Kit (Qiagen, Germany) and transformation was performed by heat shock method with *E. coli* DH5α as a recipient. The transformants were chosen on Luria Bertani agar (Hi‐Media, Mumbai, India) containing 2 µg/ml colistin. Further, validation was done both by phenotypic and by PCR analysis.

### Plasmid incompatibility typing by PCR based replicon typing

PCR based replicon typing of the plasmids carrying *mcr*-1 gene was performed for the identification of their incompatibility (Inc) types. Eighteen different replicon types such as FIA, FIB, FIC, HI1, HI2, I1/Iγ, L/M, N, P, W, T, A/C, K, B/O, X, Y, F, and FIIA were used^25^.

### Molecular characterization

Genotypic characterization was done for the screened positive isolates targeting *eptA* and *eptB* genes. Amplification for *eptA* and *eptB* genes was done by performing a PCR assay using specific oligonucleotide primers (Supplementary Table ST3). The conditions used were initial denaturation at 94 °C for 3 min followed by 35 cycles of denaturation at 94 °C for 40 s, annealing at 50 °C for 30 s, extension at 72 °C for 50 s and final extension at 72 °C for 7 min.

### Plasmid elimination assay

Plasmid elimination assay was performed for 5 isolates harboring *mcr*-1 (EC2, EC4, EC17, EC34, and EC51) in Luria–Bertani broth. The isolates were treated with sodium dodecyl sulfate (SDS) in which 2%, 4%, 6%, 8%, and 10% of SDS was added to 5 ml of LB broth, which was then inoculated with 50 µl of a bacterial suspension and kept in a shaker incubator at 37 °C for overnight incubation^26^ and 30 µl of the bacterial suspension was grown on LB agar with and without colistin. Elimination of the plasmid was estimated by calculating the number of cells grown on medium with and without colistin. Colistin resistance was used as the selective marker for plasmid curing analysis. Further, PCR analysis was performed to check whether *mcr*-1 gene was eliminated from the parent types.

### Transcriptional analysis

Expression of *eptA* and *eptB* genes was determined by quantitative real-time PCR (Applied Biosystem, USA). Oligonucleotides specific for the respective genes were used (Supplementary Table ST3). *E. coli* ATCC 25922 was used as a reference strain. All the isolates, cured mutants of *mcr*-1 harboring isolates, and transformants were inoculated in 5 ml Luria Bertani broth (Hi-media, Mumbai, India) containing 1 μg/ml of colistin and also in media without antibiotic pressure and incubated in a shaker incubator (160 rpm) at 37 ℃ for 12–16 h. Mid-log phase bacterial cultures at OD_600_ (OD value 0.4–0.5) were used for the experiment. Total mRNA was extracted by using RNeasy mini kit (Qiagen, Hilden, Germany) following the manufacturer’s instructions and reverse-transcribed into cDNA by using QuantiTect^®^ Reverse Transcription kit (Qiagen, Hilden, Germany). Quantification of *eptA* and *eptB* transcripts was performed by Picodrop (Pico 200, Cambridge, UK) and real-time PCR was performed using Power Sybr Green Master Mix (Applied Biosystem, Warrington, UK) using Step One Plus real-time detection system (Applied Biosystem, USA), and the expression level of the housekeeping gene *rpsL* of *E. coli* was used to normalize the transcriptional levels of the target genes. Further, the threshold cycle (ΔΔCt) method was employed to calculate the fold-changes in the expression of *eptA* and *eptB* in two different conditions^27^.

## Supplementary Information

Supplementary Information.

## Data Availability

The data that support the findings of this study are available from the corresponding author upon reasonable request.
