# Supplementary material for: Colistin exposure enhances expression of eptB in colistin-resistant Escherichia coli co-harboring mcr-1
Source: Sci Rep. 2022 Jan 25;12:1348. doi: 10.1038/s41598-022-05435-0 (PMC8789769; doi:10.1038/s41598-022-05435-0)
Supplement: Supplementary file 1 — Supplementary Information. [file 41598_2022_5435_MOESM1_ESM.pdf]

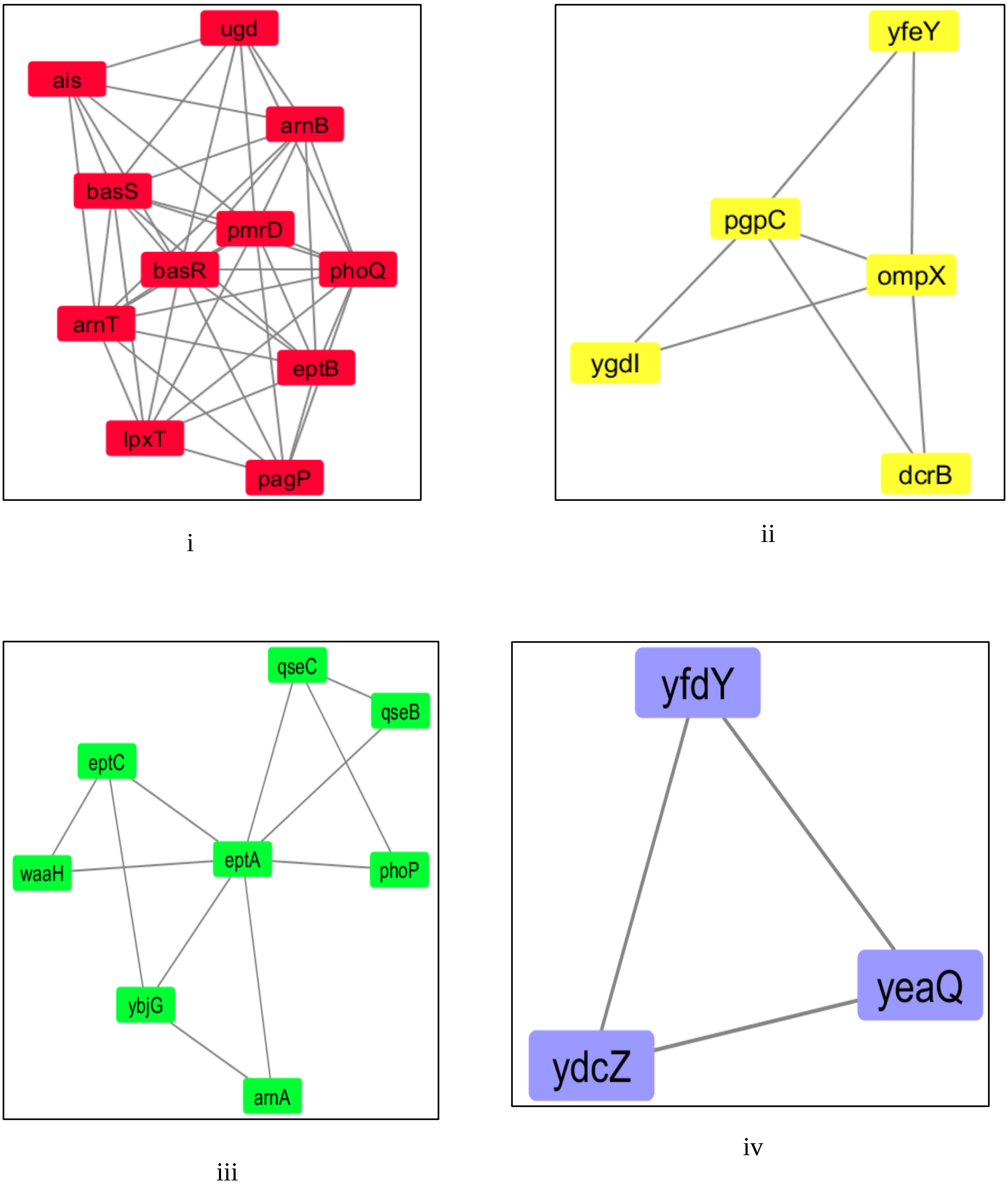

Supplementary figure S1: i. Cluster 1, ii. Cluster 2, iii. Cluster 3 and,iv. Cluster 4 of the network as generated by Cytoscape

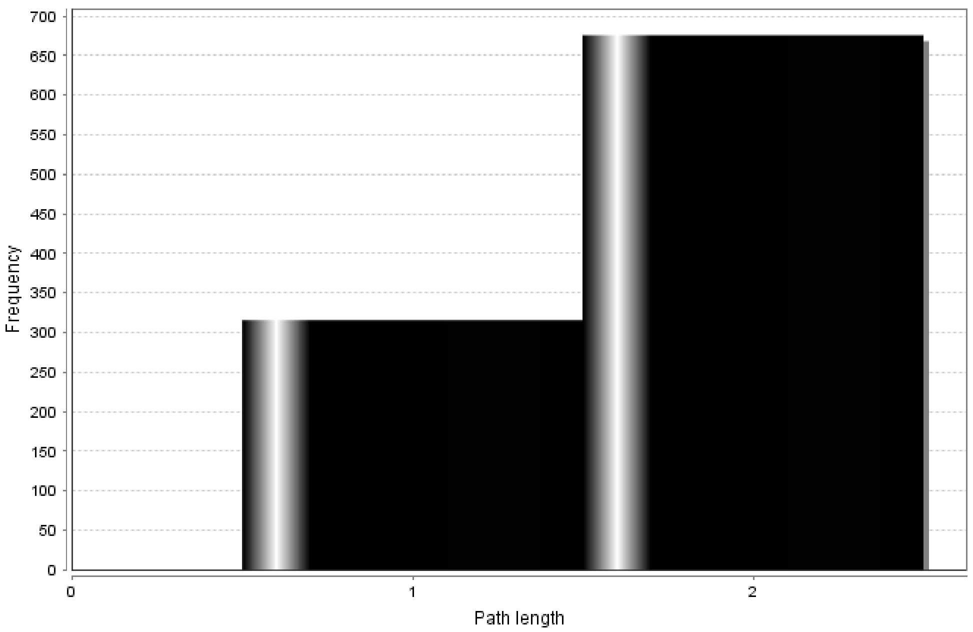

v. Average shortest path length

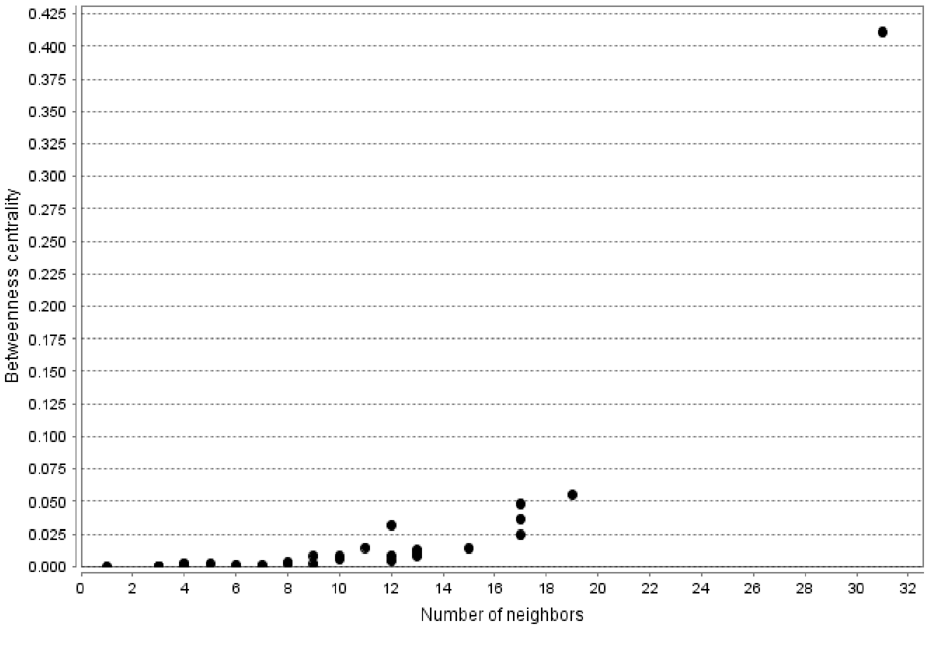

vi. Betweeness centrality

Supplementary figure S2: v and vi shows the topological parameters of the functional partners as generated by Cytoscape

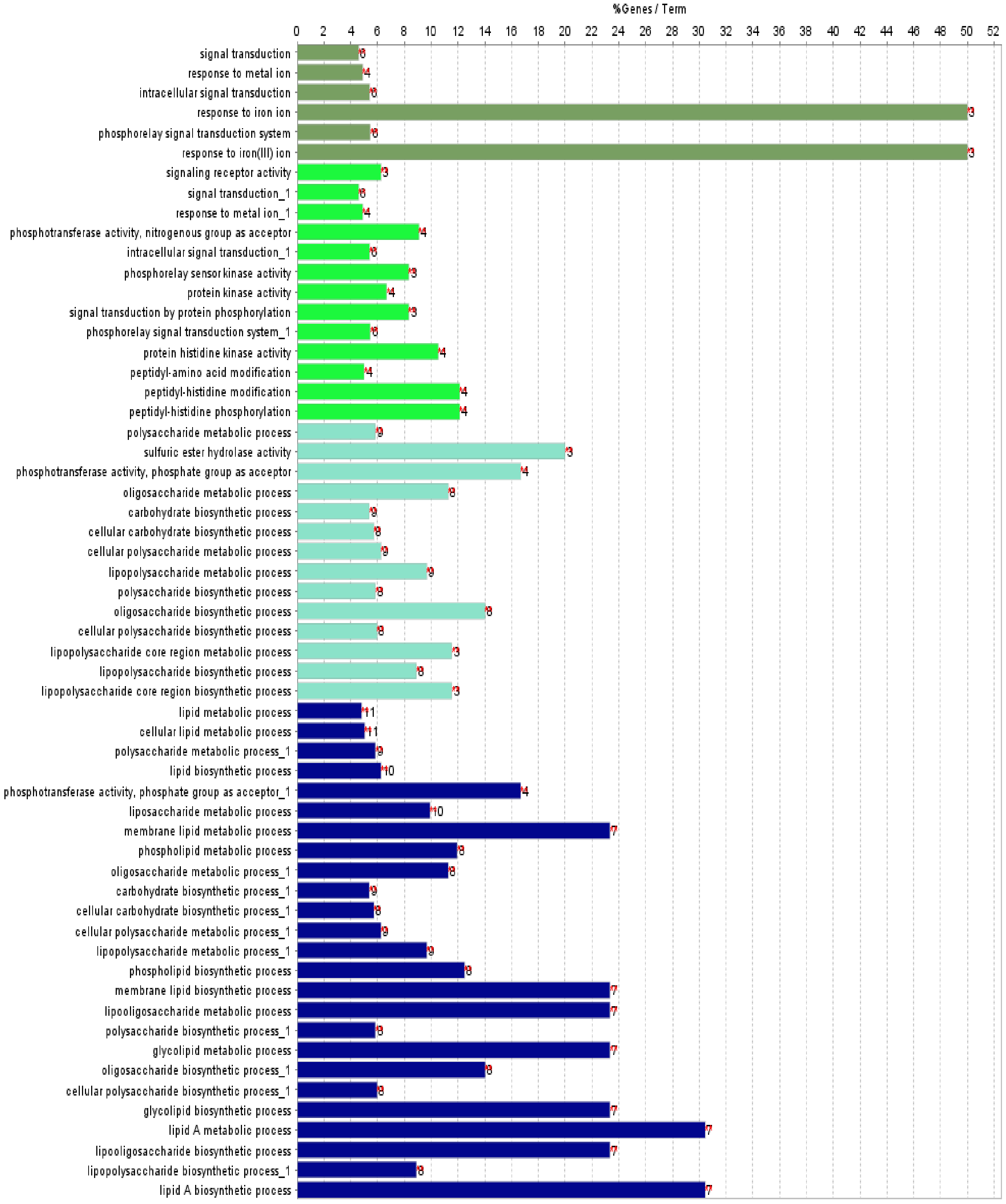

vii. Enriched GO terms associated with the functional partners

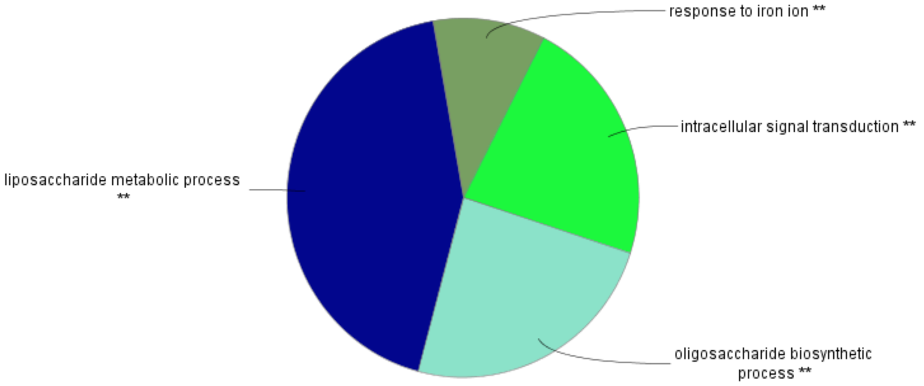

viii. Pie-chart indicates overview specific enriched clusters of the network. (Double (**) asterisk indicate significant enriched GO terms at the $p < 0.01$ statistical levels)

Supplementary figure S3: vii and viii Functional enrichment analysis of the functional partners as generated by ClueGO of Cytoscape

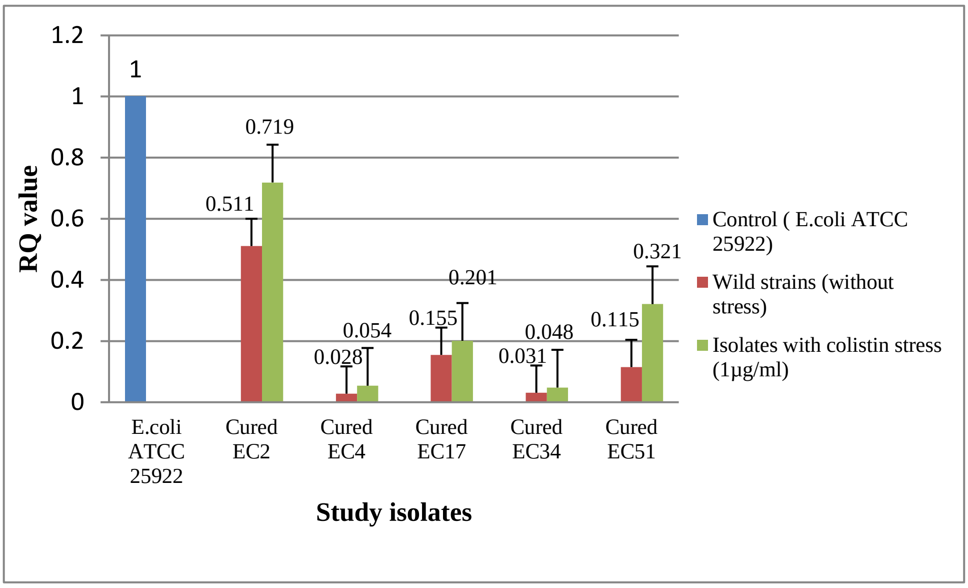

Supplementary figure S4: Transcriptional analysis of *ept*A for cured mutants

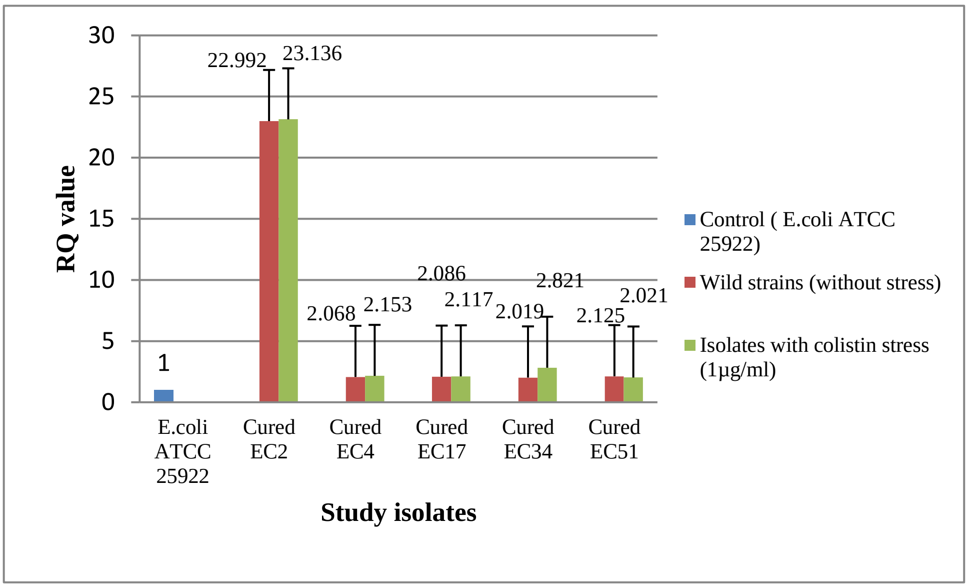

Supplementary figure S5: Transcriptional analysis of *ept*B for cured mutants

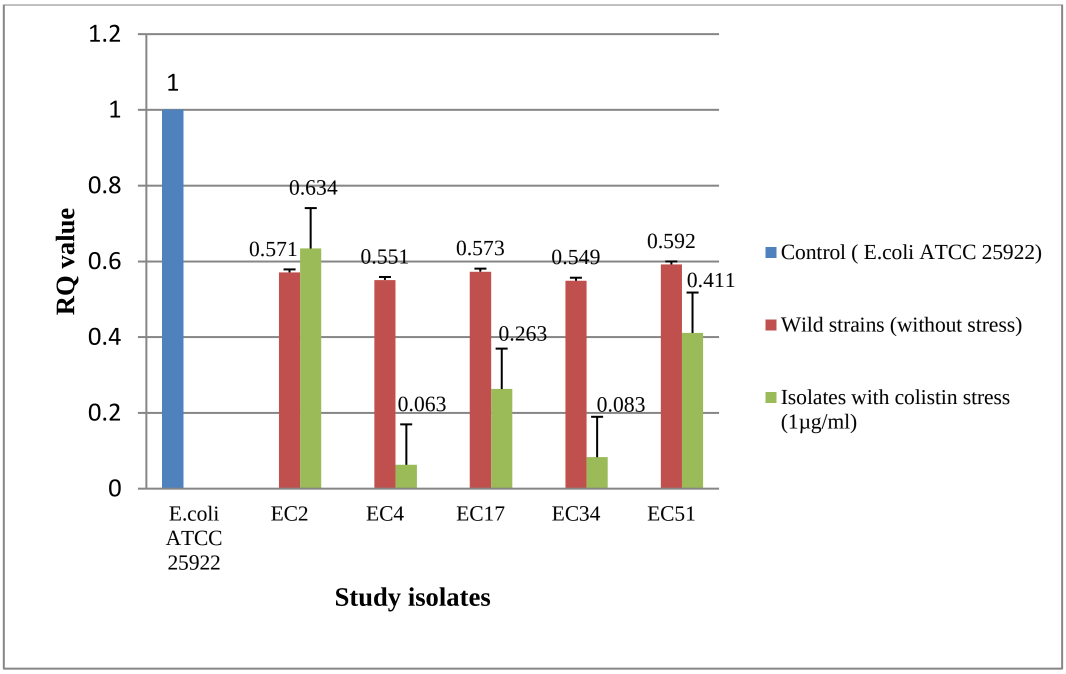

Supplementary figure S6: Transcriptional analysis of *ept*A for the transformants (in *E.coli* DH5α)

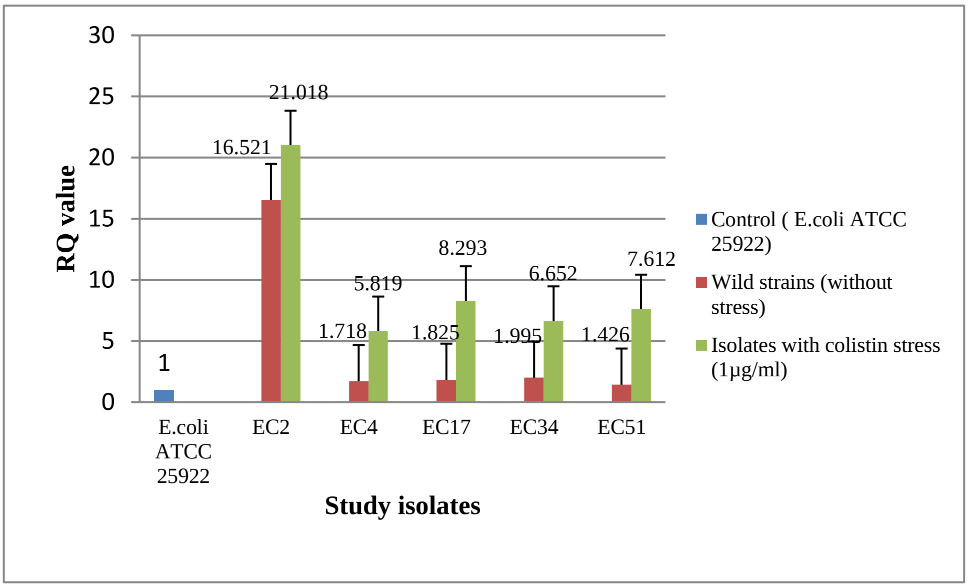

Supplementary figure S7: Transcriptional analysis of *ept*B for the transformants (in *E.coli* DH5α

Supplementary table ST1: Biological processes with which *eptB* gene is associated

Gene	Cluster	Avg. Shortest Path Length	Betweeness Centrality	Biological Processes
eptB	Cluster 1	7.418181818	1.2745098	Polysaccharide metabolic process
				Oligosaccharide metabolic process
				Carbohydrate biosynthetic process
				Cellular carbohydrate biosynthetic process
				Cellular polysaccharide metabolic process
				Lipopolysaccharide metabolic process
				Polysaccharide biosynthetic process
				Oligosaccharide biosynthetic process
				Cellular polysaccharide biosynthetic process
				Lipopolysaccharide core region metabolic process
				Lipopolysaccharide biosynthetic process
				Lipopolysaccharide core region biosynthetic process
				Lipid metabolic process
				Cellular lipid metabolic process

				Lipopolysaccharide metabolic process
				Membrane lipid metabolic process
				Phospholipid metabolic process
				Phospholipid biosynthetic process
				Membrane lipid biosynthetic process
				Lipooligosaccharide metabolic process
				Glycolipid metabolic process
				Glycolipid biosynthetic process
				Lipid A metabolic process
				Lipooligosaccharide biosynthetic process
				Lipid A biosynthetic process
				Lipid biosynthetic process

Supplementary table ST2: Trancriptional expression of *ept*A and *ept*B in the clinical isolates of *Escherichia coli*

**Sl.no.**	**Strain code**	**RQ values of *ept*A gene in reference to *E.coli* ATCC 25922 which is assigned to a value 1**		**RQ values of *ept*B gene in reference to *E.coli* ATCC 25922 which is assigned to a value 1**	
		**Wild strains**	**Strains treated with colistin**	**Wild strains**	**Strains treated with colistin**
1	ATCC	1	1	1	1
2	EC1	0.589	0.612	0.962	1.941
3	EC2	0.513	0.727	23.296	29.593
4	EC3	0.083	0.071	0.145	0.032
5	EC4	0.032	0.068	2.148	6.185
6	EC5	0.129	0.089	0.824	0.03
7	EC6	0.087	0.091	0.293	0.045
8	EC10	0.091	0.097	0.281	0.056
9	EC11	0.078	0.082	0.152	0.021
10	EC26	0.086	0.087	0.271	0.015
11	EC17	0.221	0.254	2.157	11.316
12	EC34	0.067	0.081	2.342	10.765
13	EC51	0.315	0.436	2.336	12.853
14	Cured EC2	0.511	0.719	22.992	23.136
15	Cured EC4	0.028	0.054	2.068	2.153
16	Cured EC17	0.155	0.201	2.086	2.117
17	Cured EC34	0.031	0.048	2.019	2.821
18	Cured EC51	0.115	0.321	2.125	2.021
19	Transformant (plasmid of EC2)	0.571	0.634	16.521	21.018
20	Transformant (plasmid of EC4)	0.551	0.063	1.718	5.819
21	Transformant (plasmid of EC17)	0.573	0.263	1.825	8.293
22	Transformant (plasmid of EC34)	0.549	0.083	1.995	6.652
23	Transformant (plasmid of EC51)	0.592	0.411	1.426	7.612

Supplementary table ST3: Oligonucleotides used in this study

Primer pairs	Target	Sequence(5′-3′)	Amplified product size (bp)	Reference
Epta1	*ept*A	TCTGCACGGTCTGCCTTATG	112	This study
Epta2		CAGGCAGTTCTGGTCAACCT		
Eptb1	*ept*B	AGCGTAATACCACGCCGAAA	95	This study
Eptb2		GCAACGCAGTGAGAGTTTGG		
